# Supplementary material for: Mechanical constraints to unbound expansion of B. subtilis on semi-solid surfaces
Source: Microbiol Spectr. 2023 Dec 4;12(1):e02740-23. doi: 10.1128/spectrum.02740-23 (PMC10783106; doi:10.1128/spectrum.02740-23)
Supplement: Supplemental material — Fig. S1 to S7 and Table S1. [file spectrum.02740-23-s0001.docx]

**Supplementary Information**

**
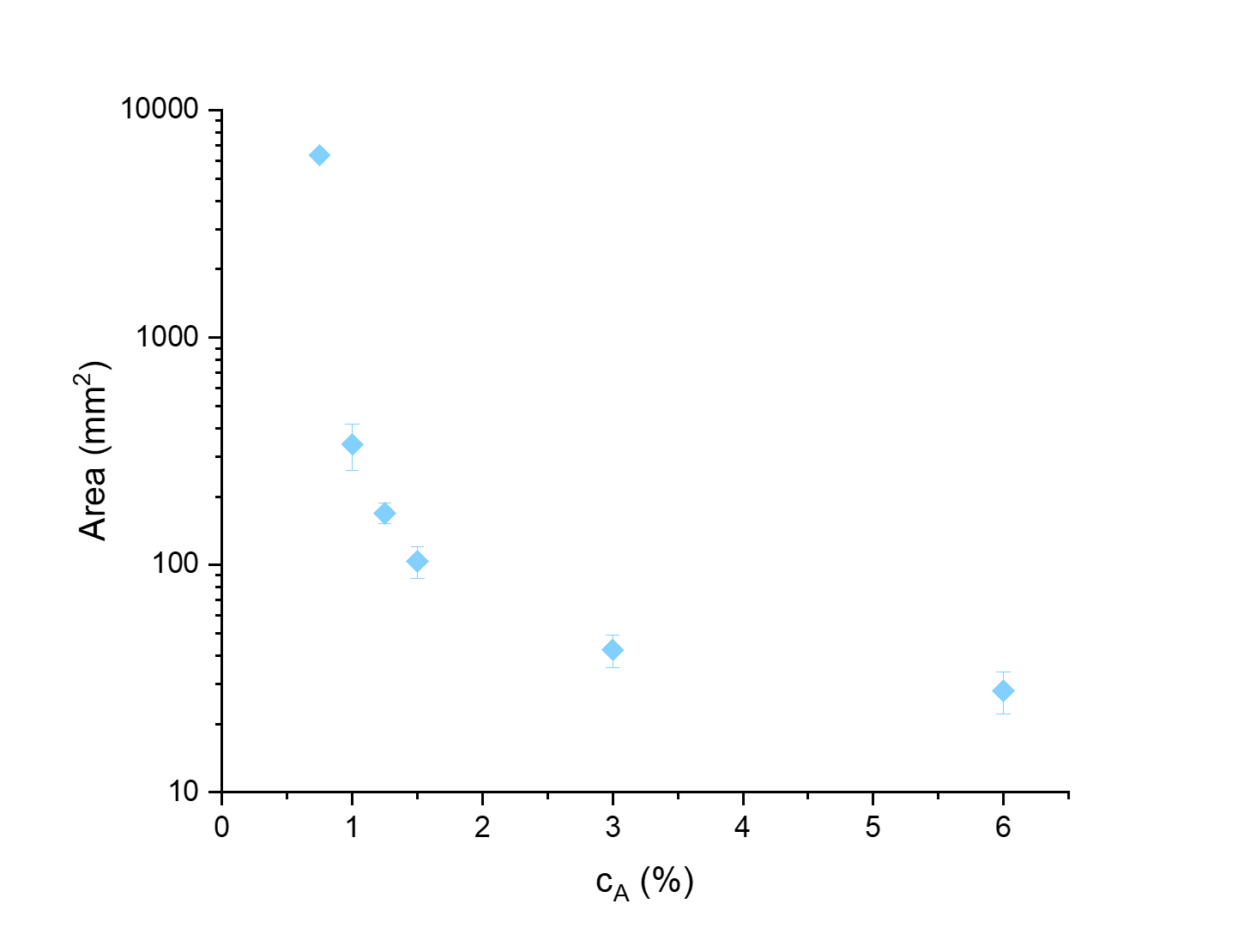
**

**Fig S1.** The extent of the bacterial expansion on different agar concentrations. The area covered by a single bacterial colony on a petri dish with a diameter of 90 mm after 20 hours of growth at 100 % nutrient concentration.


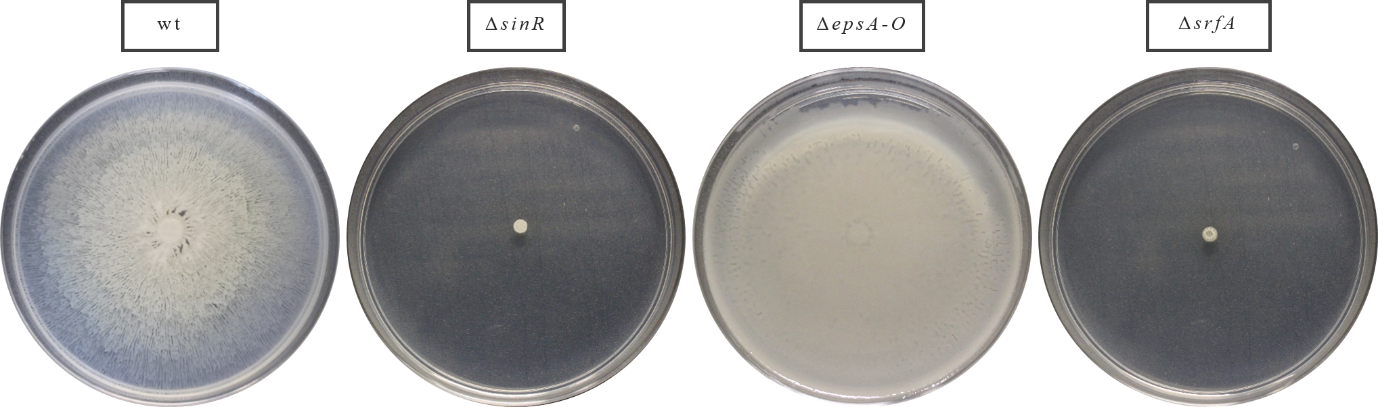


**Fig S2.** The effect of extracellular matrix components production on the expansion of *B. subtilis*. The inoculum was placed in the center of the Petri dish with a diameter of 90 mm on 0.75 % agar. Wt – wild type, Δ*sinR* – overproducer of extracellular matrix components (no bacterial expansion), Δ*epsA-O*, mutant unable to produce extracellular polysaccharide EpsA-O, Δ*srfA* mutant deficient in surfactin production (no bacterial expansion).

**Fig S3.** Viscoelastic properties of different agar gels and the corresponding biofilm properties. Biofilms were grown on agar with different agar concentration prior to the measurements. (A) Yield point marks the end of the linear viscoelastic range, (B) flow point (G′ = G″). Results are presented as mean ± standard deviation from 3-6 independent biological measurements.

**
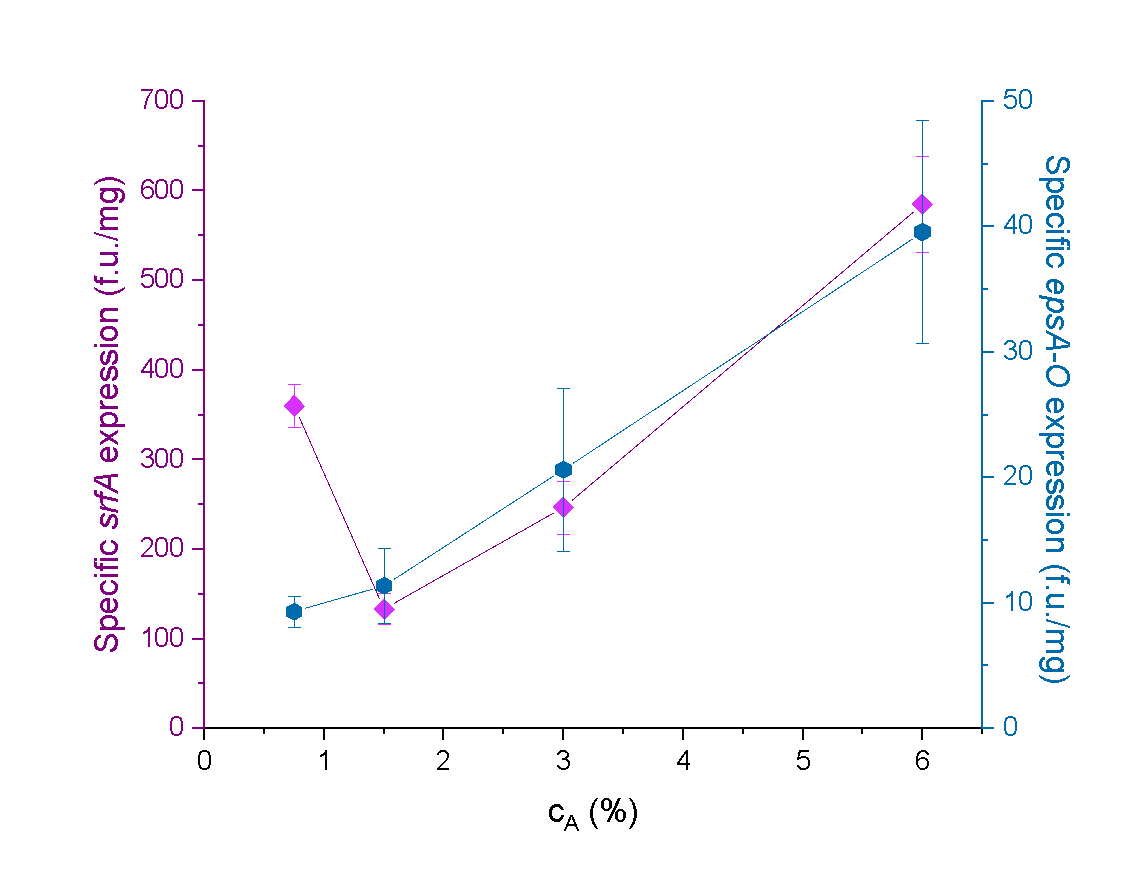
**

**Fig S4.** Normalized expression of *epsA-O* operon and *srfA* gene per bacterial mass with growth of bacteria on different agar concentrations (c_A_). Results are presented as mean ± standard deviation from 3 independent biological measurements.

**
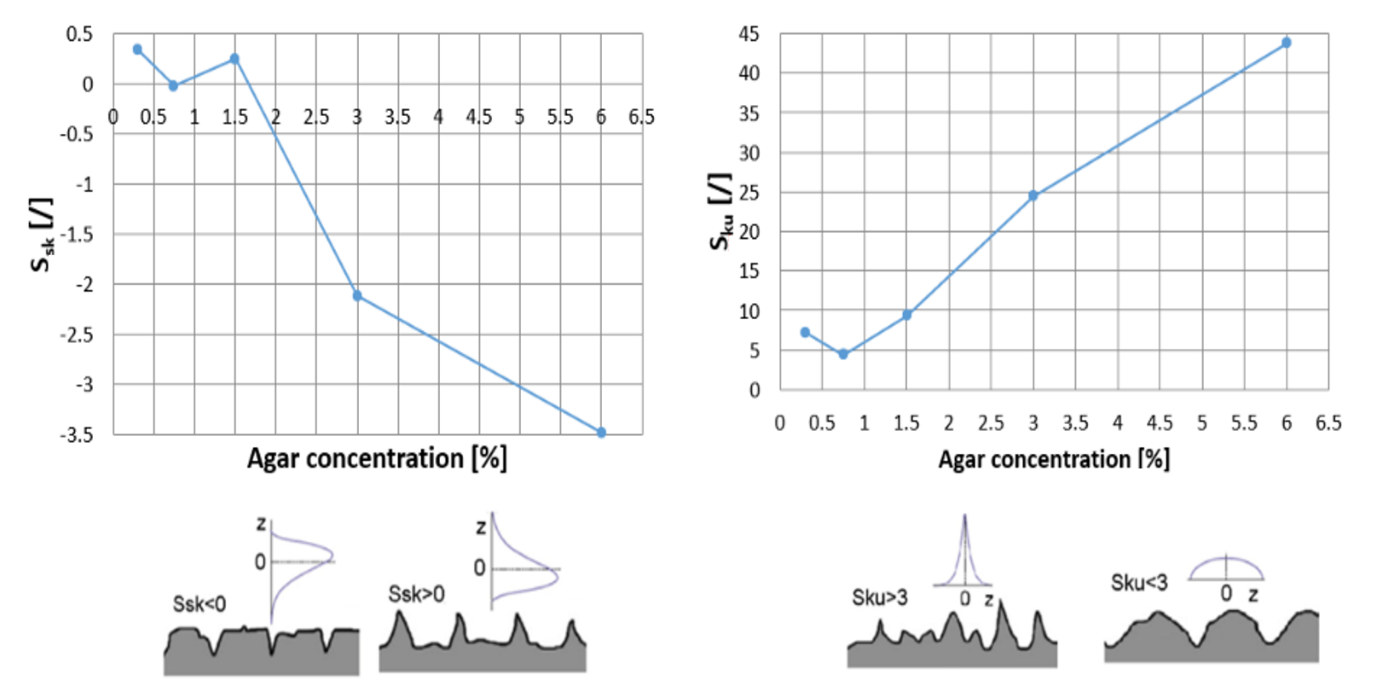
**

**Fig S5.** Skewness and kurtosis of agar plates with different agar concentrations. Skewness (Ssk) represents the degree of bias of the roughness shape (asperity). Kurtosis (Sku) is a measure of the sharpness of the asperities.


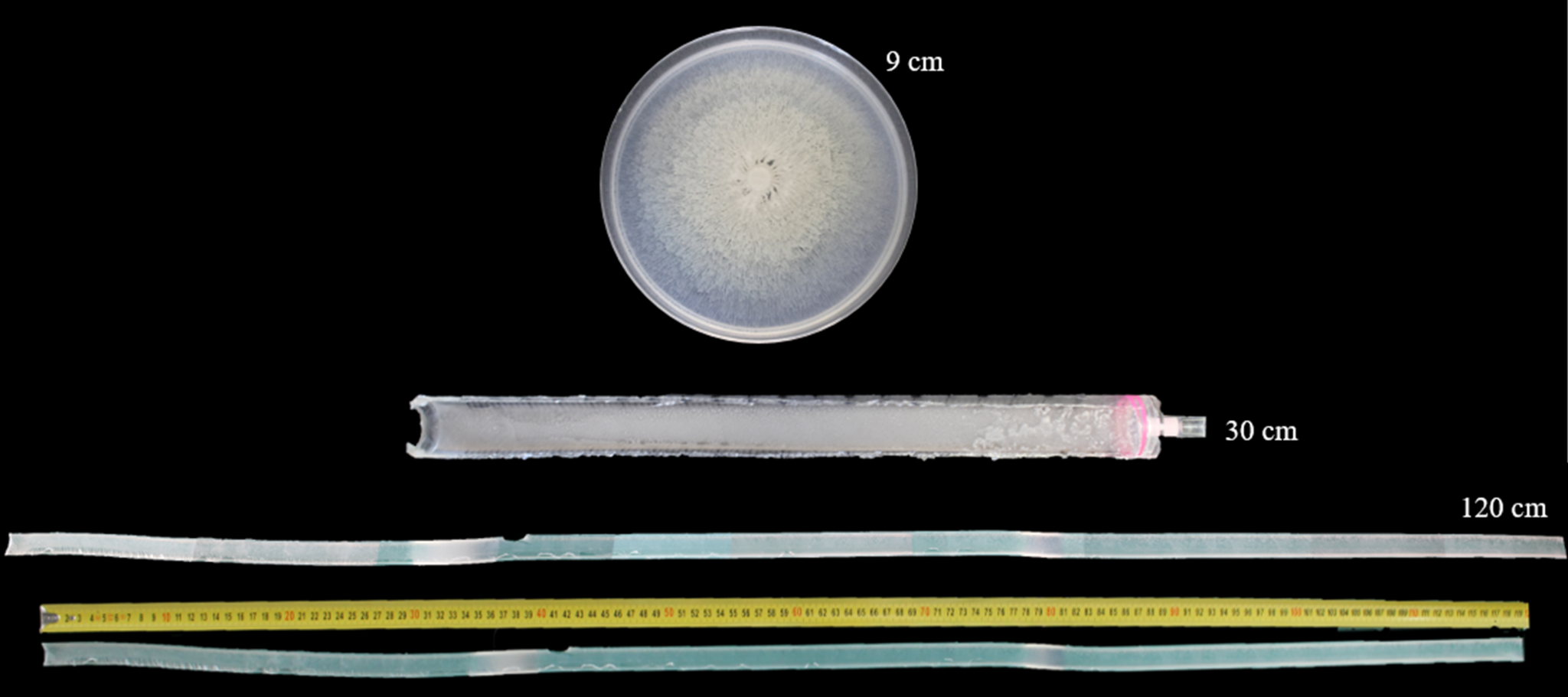


**Fig S6.** Unbounded expansion of *B. subtilis* PS-216 wt strain. *B. subtilis* was grown in different geometries on 0.75 % agar, and incubated for 20 hours at 37°C and 80 % RH. The dimensions of different geometries are indicated (not to scale). To take the photo of the long-range expansion in a 120 cm tube the agar was taken out of the tube after the incubation.

**
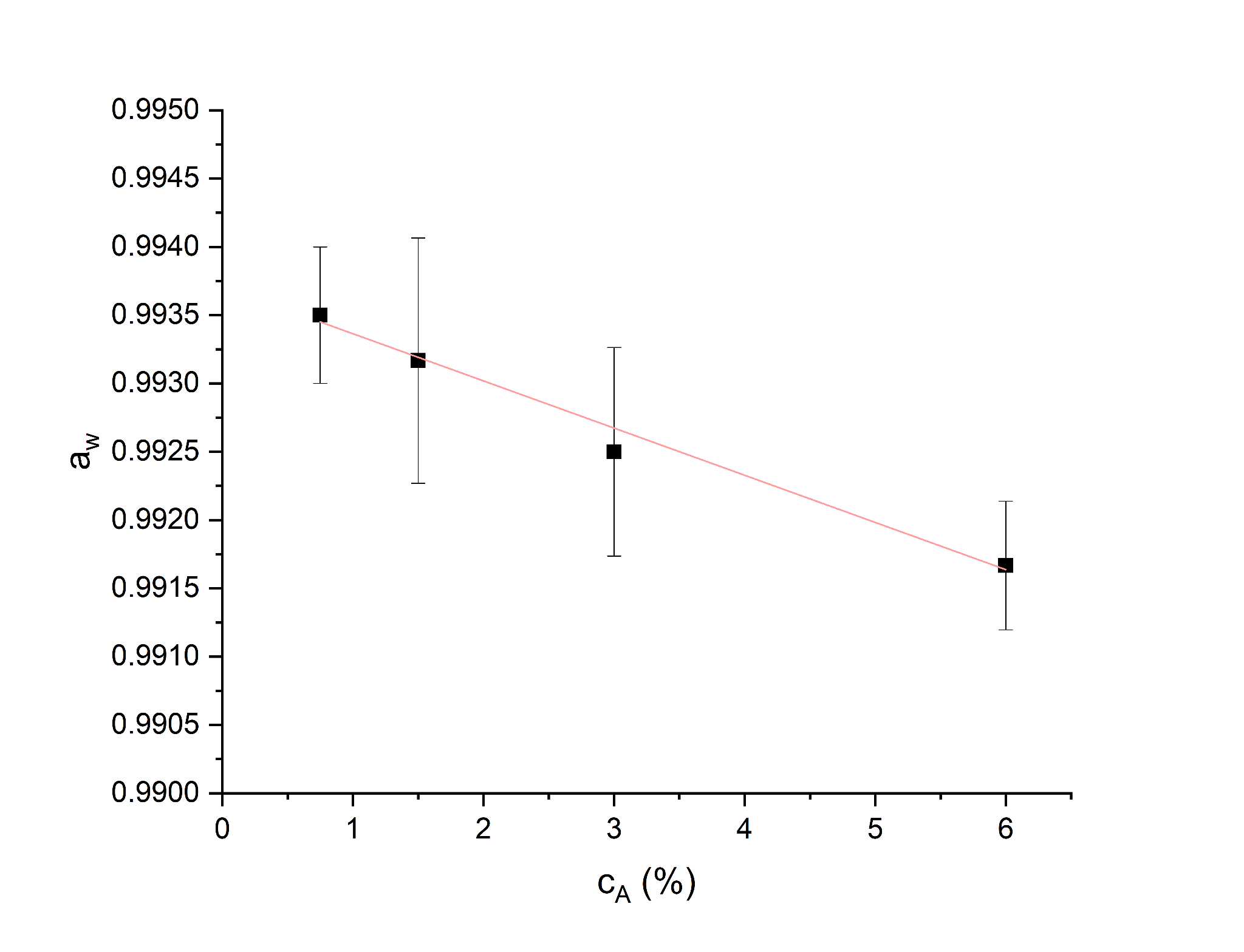
**

**Fig S7.** The effect of agar concentration (c_A_) on water activity (a_w_).

**Table S1**: *Bacillus subtilis* strains used in this study.

| ***B. subtilis* strains** | **Genetic background** | **Reference** |
| --- | --- | --- |
| **PS-216** *B. subtilis* PS-216 wt (unlabelled) | undomesticated wild type isolate | (1) |
| **ZK4300** *B. subtilis* NCIB 3610 (Δ*epsA-O*) | ∆*epsA-O::tet* (Tc) | (2) |
| **BM1070** *B. subtilis* PS-216 (Δ*epsA-O*) | ∆*epsA-O::tet* (Tc) | (3) |
| **BM1051** *B. subtilis* PS-216 (Δ*sinR*) | ∆*sinR*::*phleo* | This work |
| **BM1044** *B. subtilis* PS-216 (Δ*srfA*) | *srfA::Tn917* (Mls) | (4) |
| **DL722** *B. subtilis* NCIB 3610 (P*srfAA*) | *amyE*::P*_srfAA_*-*yfp* (Sp) | (5) |
| **BM1454** *B. subtilis* PS-216 (P*srfAA*) | *amyE*::P*_srfAA_*-*yfp* (Sp) | (6) |
| **BM1615** *B. subtilis* PS-216 (P*srfAA* P*epsA*) | *amyE*::P*_srfAA_*-*yfp* (Sp)  *sacA*::P*_epsA_*-*mKate2* (Cm) | This work |
| ***E. coli* plasmids** | **Genetic background** | **Reference** |
| pEM1089 | DH5α *sacA*::P*_epsA_*-*mKate2* (+GA) (Cm) | This work |
| **Oligonucleotide**  **Name** | **Sequence 5′→3′** | **Reference** |
| **P3F** GTACAAGCTTAAGGAGGAACTACTATGGATTCAATAGAAAAGGTAAG (6) | | |
| **P3R** GTACGGATCCTTATCTGTGCCCCAGTTTGCT (6) | | |
| **P5F** GTCGAATTCCTAGAAATTCTCCTCTATTCCTGTCG (7) | | |
| **P3R** GATCGGATCCCATAGCCTTCAGCCTTCC (7) | | |

**Supplementary References**

1. Stefanic P, Mandic-Mulec I. 2009. Social interactions and distribution of *Bacillus subtilis* pherotypes at microscale. *J Bacteriol* 191:1756–1764.

2. Lyons NA, Kraigher B, Stefanic P, Mandic-Mulec I, Kolter R. 2016. A combinatorial kin discrimination system in *Bacillus subtilis*. *Curr Biol* 26:733–742.

3. Stefanic P, Belcijan K, Kraigher B, Kostanjšek R, Nesme J, Madsen JS, Kovac J, Sørensen JS, Vos M, Mandic-Mulec I. 2021. Kin discrimination promotes horizontal gene transfer between unrelated strains in *Bacillus subtilis*. *Nat Commun* 12:3457.

4. Danevčič T, Dragoš A, Špacapan M, Stefanic P, Dogsa I, Mandic-Mulec I. 2021. Surfactin facilitates horizontal gene transfer in *Bacillus subtilis*. *Front Microbiol* 12: 1000.

5. López D, Vlamakis H, Losick R, Kolter R. 2009. Paracrine signaling in a bacterium. *Genes Dev* 23:1631–1638.

6. Špacapan M, Danevčič T, Štefanic P, Porter M, Stanley-Wall NR, Mandic-Mulec I. 2020. The ComX quorum sensing peptide of *Bacillus subtilis* affects biofilm formation negatively and sporulation positively. *Microorg* 8:1131.

7. Chai Y, Chu F, Kolter R, Losick R. 2008. Bistability and biofilm formation in *Bacillus subtilis*. *Mol Microbiol* 67:254–263.
